# Nicotinamide Riboside Opposes Type 2 Diabetes and Neuropathy in Mice

**DOI:** 10.1038/srep26933

**Published:** 2016-05-27

**Authors:** Samuel A.J. Trammell, Benjamin J. Weidemann, Ankita Chadda, Matthew S. Yorek, Amey Holmes, Lawrence J. Coppey, Alexander Obrosov, Randy H. Kardon, Mark A. Yorek, Charles Brenner

**Affiliations:** 1 Department of Biochemistry, Carver College of Medicine, University of Iowa, Iowa City, IA 52242, USA; 2 Iowa City Veterans Administration, Iowa City, IA 52246, USA; 3 Department of Opthalmology, Carver College of Medicine, University of Iowa, Iowa City, IA 52242, USA; 4 Department of Internal Medicine, Carver College of Medicine, University of Iowa, Iowa City, IA 52242, USA

## Abstract

Male C57BL/6J mice raised on high fat diet (HFD) become prediabetic and develop insulin resistance and sensory neuropathy. The same mice given low doses of streptozotocin are a model of type 2 diabetes (T2D), developing hyperglycemia, severe insulin resistance and diabetic peripheral neuropathy involving sensory and motor neurons. Because of suggestions that increased NAD^+^ metabolism might address glycemic control and be neuroprotective, we treated prediabetic and T2D mice with nicotinamide riboside (NR) added to HFD. NR improved glucose tolerance, reduced weight gain, liver damage and the development of hepatic steatosis in prediabetic mice while protecting against sensory neuropathy. In T2D mice, NR greatly reduced non-fasting and fasting blood glucose, weight gain and hepatic steatosis while protecting against diabetic neuropathy. The neuroprotective effect of NR could not be explained by glycemic control alone. Corneal confocal microscopy was the most sensitive measure of neurodegeneration. This assay allowed detection of the protective effect of NR on small nerve structures in living mice. Quantitative metabolomics established that hepatic NADP^+^ and NADPH levels were significantly degraded in prediabetes and T2D but were largely protected when mice were supplemented with NR. The data justify testing of NR in human models of obesity, T2D and associated neuropathies.

The global epidemic of obesity and diabetes has created severe economic stresses on health systems and intense neuropathic complications for affected individuals. Obesity is frequently associated with prediabetic polyneuropathy (PDPN)^1^, while about half of individuals with diabetes will suffer from diabetic peripheral neuropathy (DPN)^2^, rendering them insensitive to heat and touch. Severe DPN can progress to foot ulcers and amputations. Few treatments are effective for obesity while nothing has been found to arrest or reverse DPN. Best available care is tight glycemic control, lifestyle changes centered on dietary improvement and exercise, and pain medication when DPN is painful^3^.

Deficiency in the NAD^+^ co-enzyme causes pellagra, which was endemic a century ago in the American south in populations subsisting on corn rations and lard^4^. Though pellagra has been nearly eliminated, there are indications that supplementation with nicotinamide riboside (NR), a recently discovered NAD^+^ precursor vitamin^5^,^6^ found in milk^7^, can improve metabolic health in overfed mice^8^,^9^. Though the mechanisms accounting for resistance to weight gain and improved glycemic control for mice on high fat diet (HFD) as well as resistance to diet-induced fatty liver are not fully understood, NR elevates NAD^+^ levels in skeletal muscle, liver and brown adipose tissue and appears to increase activity of nuclear and mitochondrial NAD^+^-dependent protein lysine deacetylases including sirtuins SIRT1 and SIRT3^8^,^9^. Phosphorylated NR, *i.e.*, nicotinamide mononucleotide (NMN), also improves insulin sensitivity and glycemic control^10^ and acts as a hypothalamic activator^11^.

Degradation of axonal NAD^+^ has been implicated in Wallerian degeneration, the process by which damaged nerves die back^12^,^13^,^14^. NR but not Nam or NA protects damaged neurons, apparently because NR kinase 2 is transcriptionally induced in the damaged neuron^13^. Two mechanisms have been proposed for neuroprotection: boosting mitochondrial NAD^+^ to support SIRT3^15^, and preserving axonal NAD^+^ in the face of damage-induced SARM1 activation, which results in NAD^+^ degradation^14^. In addition, a neuroprotective mechanism has been proposed that depends on both mitochondrial and axonal NAD^+ ^16. Though NR is not only a precursor of NAD^+^ but also of NADH, NADP^+^ and NADPH^4^, the NAD^+^ metabolome has never been quantified in any disease model for which NR prevention or therapy has been tested. In addition, NR has not been tested on DPN.

Because of the potential for NR to improve prediabetic (PD) and diabetic glucose and lipid metabolism while also treating neuropathic complications, we aimed to test NR in mouse models. Thus, we used HFD to make mice obese and PD and rendered them type 2 diabetic (T2D) with HFD plus two low doses of streptozotocin (STZ)^17^. Here we show that NR improves fasting glucose levels and glucose tolerance of PD mice, while providing resistance to a substantial degree of hepatic steatosis, hypercholesterolemia, liver damage and weight gain. NR greatly lowered fasting and nonfasting glucose of T2D mice, while reducing hepatic steatosis and weight gain. Though hepatic steatosis and hyperglycemia were not fully corrected by NR, supplemented mice have greatly reduced neuropathic symptoms in both models. Remarkably, quantitative metabolomics revealed that PD and T2D mice have lower levels of hepatic NADP^+^ and NADPH, and T2D mice trended toward lower levels of hepatic NAD^+^. Upon supplementation, NAD^+^ was more correctable than was NADP^+^ and NADPH, which suggests that maintenance of levels of the latter metabolites is challenged by obesity. Our data also indicate that corneal confocal microscopy (CCM) can be used as a minimally invasive and translational assay to monitor NR-dependent improvements in PDPN and DPN in future clinical investigations.

## Results and Discussion

Sixty male C57Bl/6J mice, housed 3 or 4 per cage, were raised on Teklad 7001 normal chow (NC). At 12 weeks of age, when mice weighed ~23 g, 40 mice were transferred to HFD (Research Diets 12492, 60% calories from fat) to render them PD, while 20 mice remained on NC. After 8 weeks on HFD, 20 of 40 mice were given two low doses (75 mg/kg body weight followed 2 days later with 50 mg/kg body weight) of STZ to induce T2D. PD and T2D mice remained on HFD for the duration of the experiment. Five weeks after STZ administration to create the T2D population, 10 of 20 mice in each of the three groups (NC, HFD and HFD + STZ) were supplemented with 3 g of NR chloride per kg of their diet, thereby creating six groups of 10 mice (NC, NC + NR, HFD, HFD + NR, HFD + STZ, HFD + STZ + NR; Supplementary Fig. 1). Five weeks before sacrifice, intraperitoneal glucose tolerance tests (GTT) were performed on fasted mice. Seven weeks after the beginning of NR supplementation, one mouse from each group was sacrificed per day for 5 days per week over a 2-week period. PD mice were effectively on HFD for 21 weeks without supplementation whereas NR-supplemented PD mice were fed HFD enriched with NR for the last 8 weeks. All T2D mice were non-supplemented for five weeks post STZ administration and 10 out of 20 were supplemented with NR from week 13 to 21 on HFD. On the day of sacrifice, mice were subjected to CCM, motor neuron conduction velocity (MNCV) and sensory neuron conduction velocity (SNCV) tests, and assayed for thermal sensitivity. The remaining assays were performed post-mortem^18^.

**Figure 1:**
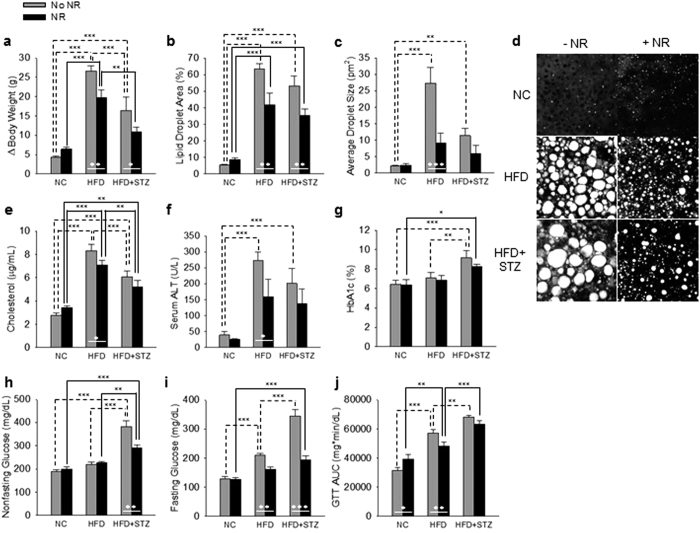
NR Improves Metabolic Parameters in PD and T2D. **(a)** NR reduces weight gain on HFD independent of STZ. **(b–d)** NR reduces hepatic steatosis in the PD and T2D models. NR lowers circulating cholesterol **(e)** and circulating alanine aminotransferase **(f)** in PD. In T2D, NR tends to lower HbA1C **(g)** and depresses nonfasting glucose **(h)**. NR depresses fasting glucose in both models **(i)**. NR improves GTT in PD **(j)**. Statistics were by two-way ANOVA. n = 10. *P < 0.05; **P < 0.01; ***P < 0.001.

As shown in Fig. 1a and Supplementary Fig. 1, during the 21 week experiment, mice on HFD gained ~27 g of body weight while mice in the HFD + STZ treatment group gained ~16 g. Though supplementation was for only 8 weeks, NR blunted weight gain in PD by ~7 g (P = 0.007) and by ~6 g in the T2D group (P = 0.031). As shown in Fig. 1b–d, mice on HFD developed severe hepatic steatosis. Whether or not HFD mice were treated with STZ, supplementation with NR strikingly reduced the hepatic oil red O-positive staining area (HFD without NR vs NR: P = 0.003; HFD+STZ without NR vs NR: P = 0.004). NR supplementation reduced oil red O droplet size by two-thirds in PD mice (P < 0.001). As shown in Fig. 1e,f, NR significantly depressed circulating cholesterol (P = 0.046) and alanine aminotransferase (ALT) (P < 0.04), a sign of liver damage, in PD mice.

As shown in Fig. 1g,h, NR tended to normalize hemoglobin A1c (HbA1c) and significantly improved nonfasting glucose (P < 0.001) in T2D. As shown in Fig. 1i, NR had a powerful effect on fasting glucose, depressing levels from 210 mg/dl to 161 mg/dl in PD mice (P = 0.008) and from 345 mg/dl to 194 mg/dl in T2D mice (P < 0.001). Finally, as shown in Fig. 1j and Supplementary Fig. 2, NR significantly improved glucose tolerance in PD (P = 0.018) and tended to improve glucose tolerance in T2D. These data indicate that NR has profound effects on whole body metabolism in PD and T2D mouse models. However, mice supplemented with NR are neither hyperactive nor hypophagic (data not shown).

**Figure 2:**
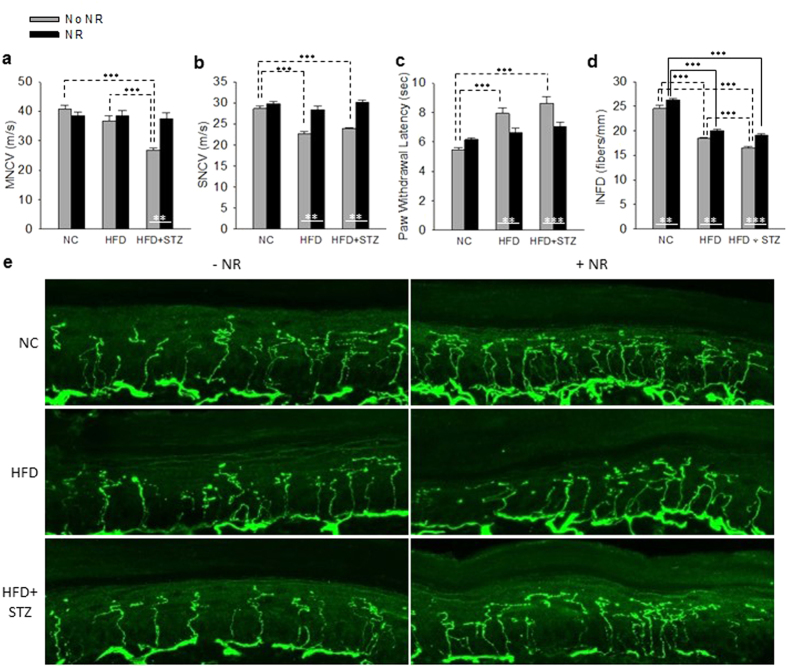
NR Opposes PDPN and T2DPN. (**a**) NR protects against a decline in MNCV in T2D. **(b)** NR protects against declines in SNCV in PD and T2D. **(c)** NR protects against loss of thermal sensitivity in both models. **(d,e)** NR improves INFD on NC and in both disease models. Statistics were by two-way ANOVA. n = 10. **P < 0.01; ***P < 0.001.

As shown in Fig. 2a,b, PD mice retained their MNCV but had significantly depressed SNCV (P < 0.001). This sensory deficit was not evident in mice supplemented with NR. T2D mice had significantly depressed MNCV (P < 0.001) and SNCV (P < 0.001) that were prevented by NR supplementation. Thermal insensitivity, a particularly dangerous aspect of human DPN^19^, was strikingly evident in the PD (P < 0.001) and T2D (P < 0.001) models and was significantly reduced by NR in PD (P = 0.003) and T2D (P < 0.001). Consistent with the sensory neuron deficits in both models, as shown in Fig. 2d,e, intraepidermal nerve fiber density (INFD) in hindpaws was significantly degraded in PD (P < 0.001) and T2D (P < 0.001). NR significantly protected against this neurodegeneration in PD (P = 0.005) and T2D (P < 0.001).

**Figure 3:**
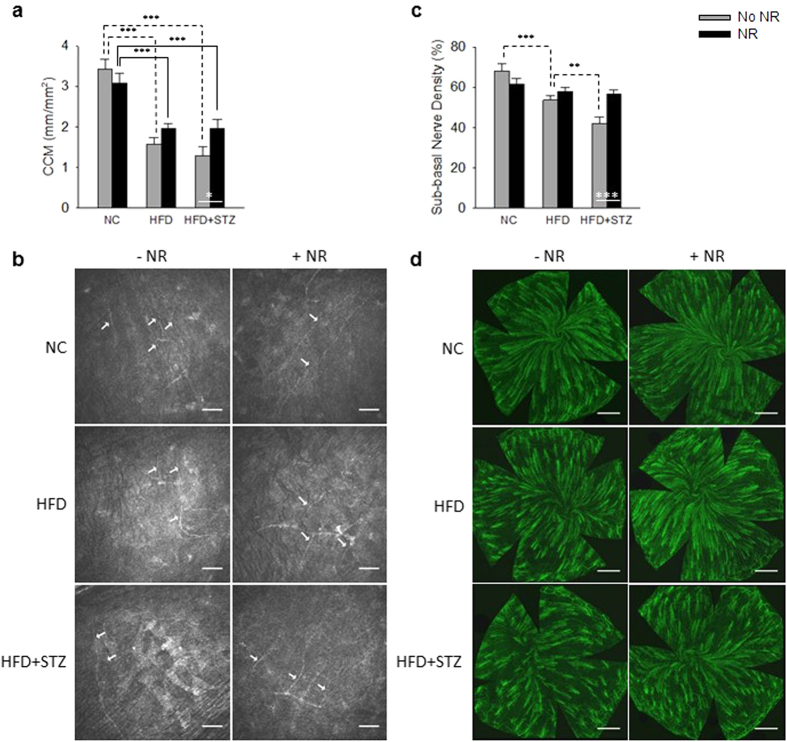
Neuroprotective Activity of NR in DPN Can be Monitored by Corneal Confocal Microscopy. **(a,b)** CCM is a sensitized assay for PD and T2D nerve loss. **(c)** and **(d)** By post-mortem class III β-tubulin staining, NR protects against corneal sub-epithelial nerve loss in T2D. Statistics were by two-way ANOVA. n = 10. *P < 0.05; **P < 0.01; ***P < 0.001.

Early small fiber neuropathic changes are difficult to quantify in human populations and this may contribute to a failure to translate potentially effective treatments from animal models of DPN to the clinic^20^. The cornea is the most densely innervated structure of the human body, containing Aδ and unmyelinated C fibers derived from the ophthalmic division of the trigeminal nerve^21^. CCM is gaining establishment as a valid measure of diabetic nerve damage in the clinic^22^,^23^ that can also be used to monitor diabetic neurodegeneration in rodent models^17^,^18^,^24^. As shown in Fig. 3a,b, quantification of sub-epithelial corneal nerves by CCM indicated that corneal nerves are severely degraded by PD (P < 0.001) and T2D (P < 0.001). CCM assays indicated that NR protects corneal innervation in T2D (P = 0.04) and tends to do so in PD. Upon sacrifice, sub-basal corneal innervation was analyzed by staining for class III β-tubulin. This assay, shown in Fig. 3c,d, produced the same qualitative results as those obtained from CCM of living mice. Thus, CCM can be used to monitor the beneficial effects of NR in T2D neuroprotection.

In cultured dorsal ganglion root neurons, the concentration of NAD^+^, as determined by LC, was reported to decline in a SARM1-dependent manner in a four hour period after axotomy^14^. Because NR affects whole body metabolism, the targets of NR supplementation are not assumed to reside in a single tissue, nor is it assumed that obesity exclusively dysregulates targets of the NAD^+^ metabolome—such as poly(ADPribose) polymerase (PARP) family members or sirtuins—that depend exclusively on NAD^+ ^25 as opposed to other NAD^+^ metabolites. Moreover, because sensory nerves die back in DPN, all neuronal metabolites are expected to fall as neuronal tissue declines with disease, such that it is difficult to normalize metabolomes in dying tissues. We hypothesized that PD and T2D might alter the NAD^+^ metabolome in multiple tissues. We therefore employed LC-MS/MS to measure the NAD^+^ metabolome on a common pmol scale^26^,^27^ in freeze-clamped liver samples from freshly euthanized mice. This technology allows one to determine whether a disease model alters NAD^+^ metabolism and the degree to which NR supplementation boosts particular metabolites. The data indicate that PD and T2D significantly dysregulate the hepatic NAD^+^ metabolome and that the center of this dysregulation is the pool of NADP^+^ and NADPH.

As shown in Table 1, liver NADP^+^ and NADPH were significantly depressed in PD and T2D (both P < 0.0001) with respect to NC controls. NADPH was also significantly depressed in T2D versus PD (P = 0.014). NR supplementation significantly boosted hepatic NADP^+^ and tended to elevate NADPH but did not fully correct either metabolite. In PD, hepatic NAD^+^ trended down (P = 0.81) and trended down further in T2D (P = 0.084) mice with respect to NC controls. Hepatic NAD^+^ was fully normalized by NR in both models—the boost in hepatic NAD^+^ achieved significance in NR-supplemented T2D mice (P < 0.027). Though hepatic NADH was not depressed in PD and T2D, NR significantly increased NADH when collapsing the PD and T2D groups (P = 0.023).

It had previously been shown that HFD produces severe hepatic lipid accumulation in mice, which primes them for loss of glycemic control with low doses of STZ^17^. Here we show that levels of liver NADP^+^ and NADPH are significantly compromised in these PD and T2D models and that NAD^+^ tends to decline in the mouse model of T2D. NR supplementation is accompanied by substantial resistance to weight gain and improvements in dyslipidemia, liver function and glycemic control in one or both models. Moreover, the PD and T2D mouse models exhibited structural and functional sensory nerve deficits that were not manifested when mice were supplemented with NR for their last 8 weeks on HFD. Though NR lowered hepatic steatosis and weight gain and greatly assisted glycemic control, NR did not normalize any of these metabolic parameters. In addition, neuroprotection cannot be explained by glycemic control alone. For example, T2D mice supplemented with NR have higher nonfasting glucose than PD mice without NR (P = 0.0012). Nonetheless, PD mice without NR have SNCV deficits, whereas T2D mice supplemented with NR do not. Thus, NR is presumed to have neuronal and hepatic targets. Finally, the decline in CCM-monitored neuronal density was more severe than any other measure of neuropathy and the protection of corneal innervation by NR was evident in the T2D model.

A large body of work has investigated NAD^+^-consuming enzymes including PARPs and sirtuins^25^. However, the SARM1-dependent factor that degrades axonal NAD^+^ in Wallerian degeneration is resistant to PARP inhibition and the pool of NADP^+^ and NADPH was not investigated^14^. Whereas NAD^+^ is the central hydride-accepting coenzyme for fuel oxidation, NADPH is the key hydride-donating cofactor for detoxification of reactive oxygen species (ROS)^28^, which is a major contributor to insulin resistance^29^. Because significant depression of NADP^+^ and NADPH occurs in PD and T2D whereas NAD^+^ only trended down and was easier to correct, we suggest that the overnutritional stresses of HFD specifically challenge maintenance of hepatic NADPH and that this is central to PD and its progression.

Cellular NADPH is known to be limited by expression of NAD^+^ kinase^30^,^31^ and could be depressed by loss of a repair system that restores damaged NADPH^32^. In addition, there are reports of an NADP^+^ phosphatase^33^ and NADP^+^ glycohydrolase activities^34^—obesity-associated induction of such enzymes could be responsible for loss of these metabolites. By diminishing levels of NADPH, any of these mechanisms could lower the capacity of hepatocytes and potentially other cells to detoxify ROS^28^ and diminish circadian functions^35^, thereby contributing to two major systems depressed in obesity. Ongoing work is designed to test the effect of NR on ROS damage in PD, T2D, PDPD and DPN, to discover the basis for depressed hepatic NADP^+^ and NADPH in PD, and to translate these results to human populations.

## Methods

### Mouse models

Mouse methods were as described with investigators blinded to treatments^17^,^18^,^19^. NR chloride was a gift of ChromaDex, Inc.

### NAD metabolomics

Measurement of ADPribose, MeNam, Me4PY, NMN, NAD^+^, NADP^+^, Nam, and NR were performed as a revision of established procedures with a Waters Acquity LC interfaced with a Waters TQD MS operated in positive ion multiple reaction monitoring mode^27^. MeNam and Me4PY were added to the analysis and detected using the following transitions: MeNam (m/z 137 > 94, cone voltage = 8 V, collision energy = 20 V); Me4PY (m/z 153 > 136, cone voltage = 24 V, collision energy = 14 V). These methods do not effectively preserve reduced co-factors. To quantify NADH and NADPH, frozen samples were sonicated in dry ice-chilled, deoxygenated, alkaline buffered, aqueous methanol. Extracts were then heated with constant shaking and centrifuged. Clarified supernatants were placed into fresh 2 ml centrifuge tubes and rested on dry ice. Pellets were re-extracted twice with the supernatants combined after each step. Samples were dried at ambient temperature using N_2_ gas. All samples were reconstituted in 0.1 ml of 50 mM ammonium acetate pH 9 immediately before analysis and placed in a Waters H class autosampler maintained at 8 °C. NADH and NADPH were separated on a Waters Acquity BEH C18 column with a gradient as described in Supplementary Methods. Analytes were detected using a Waters TQD operated in single ion monitoring, negative ion mode. Experimental samples were analyzed within 8 hours of reconstitution and quantitated using the standard addition method. Details of NADH and NADPH quantitation are included in the Supplementary Methods.

### Statistics

Data are presented as mean ± s.e.m. unless indicated otherwise. The effect of treatment, NR supplementation, and interactions of the two factors were determined by two-way ANOVA with multiple comparisons performed using the Holm-Sidak test. Time dependent measurements (*i.e.* in GTT and body weight) were analyzed across and within the six groups via two-way repeated measures ANOVA followed by Holm-Sidak tests. P-values < 0.05 were considered significant.

### Study approval

All animal procedures were approved by and carried out in accordance with guidelines of the Iowa City Veterans Administration Animal Care and Use Committee, which has an Animal Welfare Assurance (A3748–01) on file with the Office of Laboratory Animal Welfare and is fully accredited by AAALAC International.

## Additional Information

**How to cite this article**: Trammell, S. A.J. *et al*. Nicotinamide Riboside Opposes Type 2 Diabetes and Neuropathy in Mice. *Sci. Rep.* **6**, 26933; doi: 10.1038/srep26933 (2016).

## Supplementary Material

Supplementary Information

## Figures and Tables

**Table 1:** The hepatic pool of NADP^+^ plus NADPH is depressed by PD and T2D and is partially restored by NR.

	**NC**	**NC + NR**	**HFD**	**HFD + NR**	**HFD + STZ**	**HFD + STZ + NR**
NAD^+^	1200 ± 84	1500 ± 99	1000 ± 85	1200 ± 95	760 ± 72	1300 ± 180^†^
NADH	240 ± 16	300 ± 21	290 ± 13	320 ± 27	260 ± 26	290 ± 29
NADP^+^	230 ± 11	250 ± 16	150 ± 9.2^####^	200 ± 17^†,#^	150 ± 13^####^	200 ± 11^†††^
NADPH	140 ± 12	150 ± 12	100 ± 11	120 ± 8.1	*75* ± *8.3*^##^	*98* ± *10*^##^
Nam	170 ± 9.3	230 ± 20^†^	190 ± 12	180 ± 7.8^#^	130 ± 9.0	210 ± 17^††^
ADPR	84 ± 17	98 ± 19	60 ± 8.6	70 ± 14	44 ± 10	84 ± 19
MeNam	3.1 ± 0.28	4.4 ± 0.25	2.7 ± 0.14	2.9 ± 0.22	2.4 ± 0.32	3.8 ± 0.63
NMN	2.8 ± 0.13	3.7 ± 0.24	3.7 ± 0.36	3.5 ± 0.14	2.2 ± 0.30^***^	3.1 ± 0.21
Me4PY	2.6 ± 0.25	7.4 ± 0.64^†††^	6 ± 0.45^#^	8.3 ± 1.5	4.2 ± 0.37^###^	7.9 ± 0.95^†^
NR	1.2 ± 0.12	2.0 ± 0.45	1.6 ± 0.38	1.1 ± 0.15	1.0 ± 0.15	2.3 ± 1.3

^†^P < 0.05, ^††^P <0.01, ^†††^P <0.001 for effect of NR within a treatment group (*i.e.* NC vs. NC + NR, HFD vs. HFD + NR, HFD + STZ vs. HFD + STZ + NR). ^#^P < 0.05, ^####^P <0.0001 for effect of treatment versus NC within supplementation group (*i.e.* NC vs HFD, NC vs HFD + STZ, NC + NR vs HFD + NR, NC + NR vs HFD + STZ + NR). ^***^P < 0.001 for effect of STZ vs. HFD + STZ. Values are expressed as mean ± s.e.m. pmol/mg liver. Underlined concentrations within a treatment are significantly different from NC after collapsing for the effect of supplementation. Italicised results within a treatment are significantly different from HFD after collapsing for the effect of NR. The effect of treatment and supplementation of NR were analyzed by two-way ANOVA followed by multiple comparisons using the Holm-Sidak test.
