# Supplementary material for: Nicotinamide Riboside Opposes Type 2 Diabetes and Neuropathy in Mice
Source: Sci Rep. 2016 May 27;6:26933. doi: 10.1038/srep26933 (PMC4882590; doi:10.1038/srep26933)

Nicotinamide Riboside Opposes Type 2 Diabetes and Neuropathy in Mice

Samuel A.J. Trammell, Benjamin J. Weidemann, Ankita Chadda, Matthew S. Yorek, Amey Holmes, Lawrence J. Coppey, Alexander Obrosov, Randy H. Kardon, Mark A. Yorek and Charles Brenner

Supplementary Methods

Sample Extraction for NADH and NADPH

Portions of the same frozen, pulverized liver samples used in standard NAD metabolomic analyses were aliquoted (19 – 29 mg) into liquid N_2-cooled 2 ml centrifuge tubes and stored at -80 °C until analysis. Prior to extraction, extraction solvent (5 parts 100% methanol:1 part 25 mM ammonium acetate pH 9) was sparged with N_2 gas to remove oxygen. Samples were extracted by addition of 0.5 ml of extraction solvent at dry ice temperature. Samples were sonicated (Branson 450 Sonifier, Danbury, CT, USA) at an output control of 4 and intensity of 40% for 10 minutes in an acetone/water bath cooled to -4 °C. Samples were returned to dry ice until all had been processed. Samples were then heated at 60 °C for 3 minutes with constant shaking at 1050 rpm using a Thermomixer® (Eppendorf, Hamburg, Germany). Samples were centrifuged (16.1 k*g*, 4 °C, 10 minutes) and clarified supernatants were transferred to fresh, dry ice-chilled 2 ml centrifuge tubes. Two repeated extractions were performed on the pellets. All clarified supernatants were combined into the same 2 ml centrifuge tubes. An additional 70 mg of frozen, pulverized liver was aliquoted between three 2 ml centrifuge tubes frozen at liquid N_2 temperatures. These aliquots were treated in the same manner as the samples except that clarified supernatants from all three tubes were combined into the same dry ice-chilled 15 ml centrifuge tube. After all extractions had been completed, extract equivalent to 5 mg of liver was aliquoted into nine fresh 2 ml centrifuge tubes. NADH (N1161, Sigma Aldrich, St. Louis, MO, USA) and NADPH (N0411, Sigma Aldrich) were suspended in 25 mM ammonium acetate pH 9 immediately prior to analysis of each batch of samples. Concentration was determined using extinction coefficients (340 nm) of 6,220 $M^{-1} * cm^{-1}$ for each analyte. Stocks were diluted to 0.5 mM in the same solution then diluted to produce a

standard curve (45, 75, 100, 150, 250, 300, 375 µM). 10 µl of standard was transferred to a tube containing extract equivalent to 5 mg of liver. Liver extract containing standard and liver sample were dried at ambient temperature using N_2 gas. Prior to LC-MS analysis, all samples were reconstituted in 100 µl of 50 mM ammonium acetate (>99% pure) pH 9 in LCMS-grade water. Samples were transferred to Waters polypropylene plastic total recovery vials (Part # 186002639) and stored in a Waters Acquity H class autosampler maintained at 8 °C until injection at either 2.5 or 10 µl for samples and standards, respectively. Samples were analyzed within 8 hours.

LC-MS Analysis for NAD(P)H

Chromatographic separation was performed using a 2.1 mm X 100 mm Waters Acquity BEH C18 column and separated using a gradient. Mobile phase A contained 10 mM triethylammonium acetate and 15 mM acetic acid. Mobile phase B was composed of LCMS grade methanol. Flow rate was set at 0.4 ml/min and separation was performed using a gradient with initial equilibration at 0% B, held for 1.25 minutes, a gradient over 1.25 minutes to 20% B and held for 3.25 minutes, followed by a gradient over 2.75 minutes to 55% B, then a gradient over 1.5 minutes to 95% and held for another 1.5 minutes, then reset to initial conditions and held for 4.2 minutes. The column was maintained at 25 °C. Analytes were detected and quantified using a Waters TQD operated in negative ion single ion mode. Samples were electrospray-ionized at a capillary voltage of – 2.5 kV, a desolvation gas flow rate of 700 l/hr, a cone gas flow rate of 100 l/hr, a desolvation temperature of 2350 °C, and a source temperature of 150 °C. Cone voltage was set to 20 and 52 V for NADH and NADPH, respectively. Analytes were quantified using a calibration curve and the standard addition method. All pmol amounts were normalized to g of wet liver weight extracted. Any samples outside of the calibration curve were re-extracted and analyzed.

Supplemental Figure 1. Experimental Design and Weight Gain

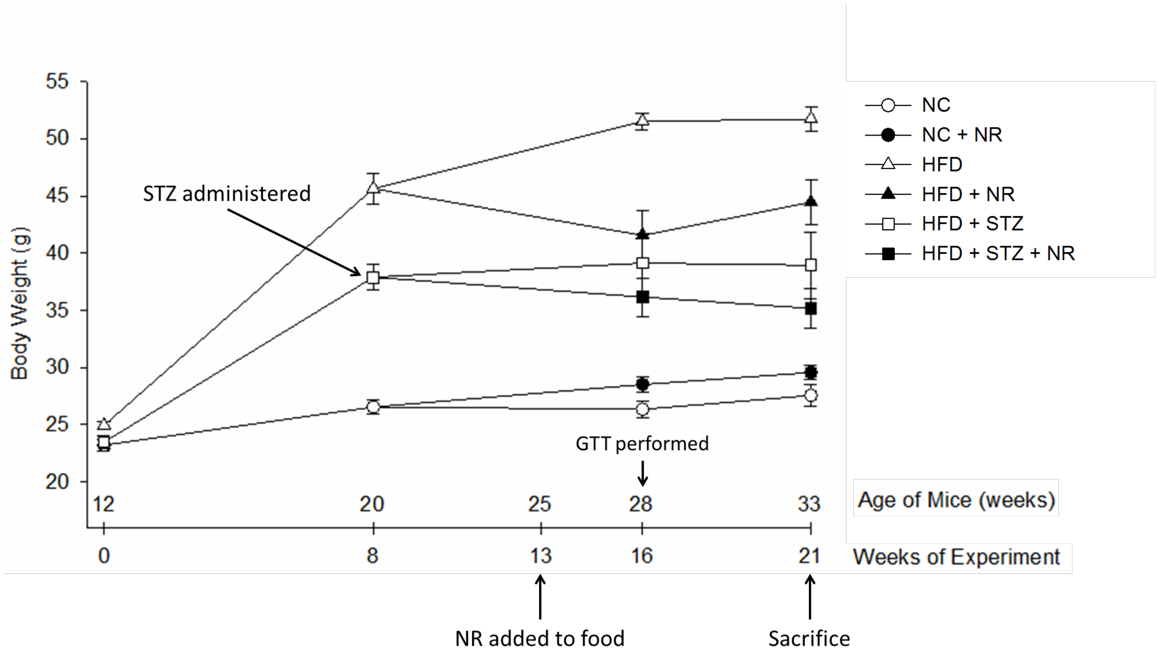

Supplemental Figure 2. GTT Primary Data

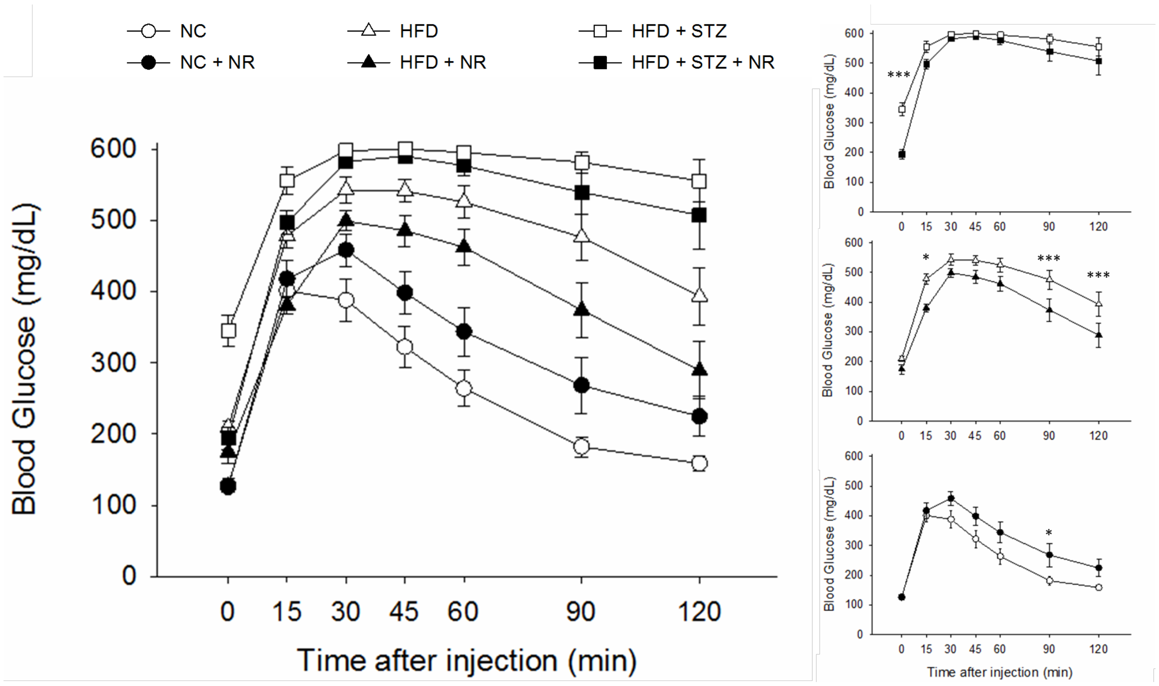

Supplement: Supplementary Information [file srep26933-s1.pdf]
